# Incidence and factors related to nonmotorized scooter injuries in New York State and New York City, 2005–2020

**DOI:** 10.1186/s12889-022-14302-6

**Published:** 2022-10-27

**Authors:** Peter Tuckel

**Affiliations:** Department of Sociology, Hunter College, City University of New York, 695 Park Avenue, New York, NY 10065 USA

**Keywords:** Nonmotorized scooters, Unpowered scooters, Kick scooters, Injuries, Epidemiology, Emergency department

## Abstract

**Background:**

This study provides an analysis of contemporary trends and demographics of patients treated for injuries from nonmotorized scooters in emergency departments in New York state excluding New York City (NYS) and New York City (NYC).

**Methods:**

The study tracks the incidence of nonmotorized scooter injuries in NYS and NYC from 2005 to 2020 and furnishes a detailed profile of the injured patients using patient-level records from the Statewide Planning and Research Cooperative System (SPARCS). A negative binomial regression analysis is performed on the SPARCS data to measure the simultaneous effects of demographic variables on scooter injuries for NYS and NYC. The study also examines the demographic correlates of the rate of injuries at the neighborhood level in NYC. A thematically shaded map of the injury rates in New York City neighborhoods is created to locate neighborhoods with greater concentrations of injuries and to identify the reasons which might account for their higher rate of injuries, such as street infrastructure.

**Results:**

In NYS and NYC injuries from unpowered scooters underwent an overall decline in the past decade. However, both NYS and NYC are now evidencing an increase in their rates. The upswing in the rate in NYC in 2020 is particularly noticeable. Males and children in the age group 5 to 9 were found to be most susceptible to injury. Injuries were more prevalent in more affluent New York City neighborhoods. A map of the injury rates in the City’s neighborhoods revealed a clustering of neighborhoods with higher than average injury rates.

**Conclusions:**

Injuries from nonmotorized scooters number approximately 40,000 annually in the US and can be prevented by greater use of protective equipment. Street infrastructure is a critical factor contributing to injuries from the use of nonmotorized scooters. Thematically shaded maps can be used to identify and target areas for purposes of intervention.

## Background

With the advent of electric scooters or e-scooters, epidemiologic study has shifted away from injuries owing to nonmotorized scooters. Little systematic study has been accorded this topic in the last decade. Yet it is estimated that approximately 40,000 individuals are injured from using a nonmotorized scooter each year in the United States [1]. The epidemiologic research which has been undertaken concerning nonmotorized scooters generally has focused on individual-level attributes of patients, their diagnoses, and treatment modalities [2–5].

This study provides an analysis of contemporary trends and demographics of patients treated in emergency departments for nonmotorized scooter injuries in New York state excluding New York city (NYS) and New York city (NYC). The study tracks the incidence of patients injured from the use of nonmotorized scooters from 2005 to 2020 and describes the demographic characteristics of patients in NYS and NYC. In addition, the analysis investigates the demographic correlates of the rate of injuries from the use of nonmotorized scooters in each of the neighborhoods in NYC and maps the incidence of the injury rate in the different neighborhoods to identify patterns of geographic concentration. Thus the analysis examines the effect of both individual-level and contextual-level variables on the risk of injury.

## Methods

### Data sources

The author analyzed data primarily from emergency department (ED) visits for NYS and NYC. The analysis centered on patient-level records for NYS and for NYC consisting of a wide number of demographic, diagnostic, and treatment variables. Geographic identifiers such as the 5-digit zip code in which the patient lives were also included among the variables in these records.

The patient-level records were accessed from the Statewide Planning and Research Cooperative System (SPARCS) [6]. SPARCS is responsible for maintaining information on all outpatient, inpatient, and ambulatory surgery patients treated in a hospital located in the state of New York.

### Variables

#### Injury code

Two separate injury codes provided identification of patients who were injured while using a nonmotorized scooter. The specific codes used in this study were restricted to patients who fell from a nonmotorized scooter. The International Classification of Diseases, Ninth Revision (ICD-9-CM) External Cause of Injury Code (E-code) E885.0 – Fall from (nonmotorized) scooter – was used for the years prior to 2015. On October 1, 2015 ICD-9-CM was replaced by ICD-10-CM. Therefore both the ICD-9-CM E-code 885.0 and the ICD-10-CM code V00.141A – Fall from (nonmotorized) scooter, initial encounter – were applied for the year 2015. However, only the ICD-10-CM code V00.141A was applied for the years from 2016 to 2020.

#### Sociodemographic characteristics

In addition to the SPARCS data providing information about the age and gender of patients, SPARCS also included two variables relating to the patient’s race and ethnicity. These two variables were used to construct a typology of race-ethnicity consisting of 4 values: non-Hispanic White, non-Hispanic Black, non-Hispanic Asian, and Hispanic.

### Statistical analyses

Two generalized linear negative binomial regression analyses with log-link (NB2 models) were performed to measure the total effects of year and demographic characteristics (i.e., gender, age, racial-ethnic background) on the incidence of injuries resulting from falling from a nonmotorized scooter, The first analysis was conducted among patients residing in NYS. The second analysis was restricted to patients residing just in NYC. Negative binomial regression analyses were performed instead of Poisson regression because of the presence of overdispersion in the data.

The dependent variable in these analyses consisted on the population-based counts of the number of outpatients and inpatients together who sustained an injury due to a fall from a nonmotorized scooter. The predictor variables were year, year squared, year cubed, and the patient’s gender, age, and racial-ethnic background. Year was measured as an interval-level variable with values ranging from 1 (corresponding to the year 2005) to 16 (corresponding to the year 2020). Year squared and year cubed terms were inserted in the analysis to measure any nonlinear effects of the time variable. Gender was coded by a value of 1 for male and 2 for female. Age consisted of 6 categories: under 5, 5 to 9, 10 to 14, 15 to 24, 25 to 44, and 44 and older. The racial-ethnic variable was composed of 4 groups: non-Hispanic White, non-Hispanic Black, non-Hispanic Asian, and Hispanic (any race).

An offset variable was introduced into both analyses to control for the differing risk levels of a scooter injury associated with varying population sizes, The offset variable was created via a two-step process. First, population counts (based on the Centers for Disease Control and Prevention’s Bridged-Race Population Estimates, 1990–2020) were derived for each combination of year, gender, age-group, and racial-ethnic category separately for NYS and for NYC [7]. As an example one population count would consist of non-Hispanic Black females between the ages of 10 to 14 living in NYC in 2014. Altogether, the total number of population counts equaled 768 each for NYS and for NYC.

A multiple-step procedure was undertaken to measure the demographic variables associated with the rate of scooter injuries at the neighborhood level in NYC, Step 1: The number of outpatients and inpatients combined under the age of 18 were summated for each 5-digit zip code in NYC with a nonzero population (*N* = 179) for the years 2018, 2019, and 2020. Step 2: These numbers were averaged across the three years. Step 3: The averages were aggregated up to the United Health Fund (UHF) level (*N* = 42) and divided by the population of each UHF district estimated to be under the age of 18 to obtain an injury rate. The injury rates were then correlated with a battery of socio-demographic variables originally tabulated at the zip code level which were also aggregated up to the UHF level. The socio-demographic variables were derived from the American Community Survey (ACS) 2005–2019 (5-Year Estimates) [8]. The following variables were used: (1) the racial-ethnic composition of the UHF district, (2) median family income, (3) per capita income, (4) percent of the population 25 years of age and over with a B.A. degree or more, (5) percent of the population under the age of 18 below the poverty rate, (6) percent of the population without health insurance, and (7) percent of those with health insurance who have public health insurance.

An additional analysis was also undertaken to determine if there were a relationship between the presence or absence of a skate park and the injury rate. A list of the “official” and other major skate parks in NYC (*N* = 37) was employed to carry out this analysis. An indicator variable was then created with values of 1 and 0 to measure the presence or absence of a skate park in NYC zipcodes. These data were then aggregated up to the UHF district level.

### Spatial analysis

A spatial analysis was carried out to identify the existence of geographic patterns of concentration in the incidence of falls from unpowered scooters at the neighborhood level in NYC. This analysis consisted of creating a thematically shaded map of the injury rate by the UHF district in which the patient lived. A Global Moran’s I was calculated to uncover any significant clustering in the spatial distribution of patients’ residences.

## Results

### Overall trends

**Fig. 1:**
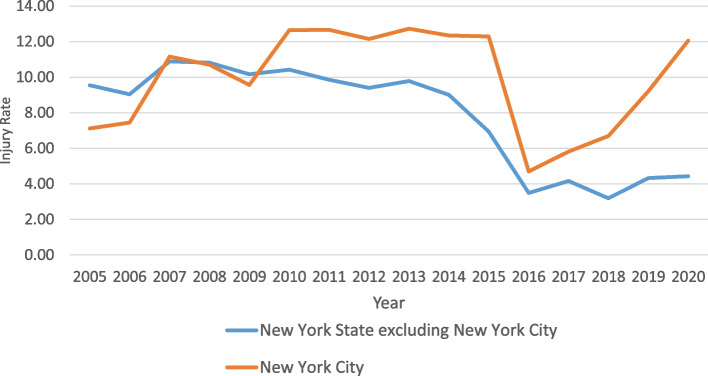
Annual Injury Rate from Nonmotorized Scooters (per 10,000) by New York State Excluding New York City and New York City

Figure 1 depicts the annual rate of injuries due to falls from nonmotorized scooters in NYS excluding NYC and NYC during the time span from 2005 to 2020. For NYS excluding NYC, the rate of injuries veered upwards from 2005 toto 2008, declined moderately from 2008 to 2014, underwent a precipitous fall in 2015 and inched up slightly since then. For NYC the rate climbed from 2005 to 2010, plateaued until 2014, sharply declined in 2015, and then has spiraled upwards from 2016 onwards.

### Demographics and other characteristics

In line with previous research findings, both gender and age are strongly related to the incidence of injury [2, 3, 5]. The injury rate of males is more than 1.6 times the corresponding rate for females (Table 1). With respect to age, the highest rate is among the age group 5 to 9 (50.45), followed by the age group 10 to 14 (35.13), and then children under 5 (14.23). The rate of injuries declines sharply after the age of 14. Overall, the rate of Hispanics (8.74) is somewhat greater that of non-Hispanic whites (7.09) and non-Hispanic blacks (7.93). These three groups exceed by a wide margin the rate of non-Hispanic Asians (4.27).

**Table 1:** Demographics and rates of patients treated for nonmotorized scooter related injuries: 2005-2020^a^

Characteristic	New York State		
	Excluding		
	New York City	New York City	Total
	Number (Rate)	Number (Rate)	Number (Rate)
**Total**	**12,285**	**10,278**	**22,563**
Gender			
Male	7100 (8.15)	6521 (10.55)	13,621 (9.14)
Female	5185 (5.74)	3757 (5.51)	8942 (5.64)
Age group			
Under 5	1014 (10.24)	1591 (18.94)	2605 (14.23)
5–9	5047 (47.57)	4057 (54.56)	9104 (50.45)
10–14	4171 (36.52)	2417 (32.97)	6588 (35.13)
15–24	740 (2.98)	657 (3.88)	1397 (3.34)
25–44	566 (1.31)	878 (2.15)	1444 (1.72)
45 and older	747 (.96)	678 (1.38)	1425 (1.13)
Race-Ethnicity			
Non-Hispanic White	9492 (6.95)	3353 (7.52)	12,845 (7.09)
Non-Hispanic Black	1253 (7.79)	2470 (8.0)	3723 (7.93)
Non-Hispanic Asian	180 (2.55)	910 (4.92)	1090 (4.27)
Hispanic	1360 (7.45)	3545 (9.37)	4905 (8.74)

^a^Rates calculated per 100,000 population

### Combining trends and demographics

Table 2 exhibit the results of two negative binomial regression analyses examining the simultaneous effects of year, the nonlinear effects of year, and demographic variables on the incidence of scooter injuries resulting in a visit to a hospital ED.

**Table 2:** Negative Binomial Estimates of Injuries From Nonmotorized

Variable	b	Exp (b)	*p* value	95% CI
New York State (excluding New York City)				
Year	.373	1.452	.001	1.175-1.795
Year squared	-..050	.951	.001	.924-.979
Year cubed	.002	1.002	.004	1.001-1.003
Gender				
Male	.418	1.519	.000	1.283-1.799
Female	(ref. cat.)			
Age category				
Under 5	2.297	9.940	.000	7.291-13.552
5 to 9	3.788	44.177	.000	32.872-59.370
10 to 14	3.339	28.187	.000	20.956-37.912
15 to 24	.984	2.675	.000	1.951-3.668
25 to 44	.295	1.343	.070	.977-1.848
45 and older	(ref. cat.)			
Race/ethnicity				
Non-Hispanic White	.243	1.275	.029	1.025-1.586
Non-Hispanic Black	.253	1.288	.031	1.023-1.622
Non-Hispanic Asian	-.791	.453	.000	.343-..599
Hispanic	(ref. cat.)			
New York City				
Year	.498	1.645	.000	1.353-2.001
Year squared	-.068	.934	.000	.910-.959
Year cubed	.003	1.003	.000	1.002-1.004
Gender				
Male	.673	1.960	.000	1.681-2.286
Female	(ref. cat.)			
Age category				
Under 5	2.583	13.238	.000	10.116-17.323
5 to 9	3.790	44.246	.000	33.918-57.718
10 to 14	3.206	24.674	.000	18.889-32.231
15 to 24	.968	2.632	.000	1.995-3.473
25 to 44	.416	1.516	.003	1.153-1.994
45 and older	(ref. cat.)			
Race/ethnicity				
Non-Hispanic White	.146	1.158	.173	.938-1.429
Non-Hispanic Black	.008	1.008	.941	.815-1.246
Non-Hispanic Asian	-.464	.629	.000	.504-.785
Hispanic	(ref. cat.)			

*Abbreviation*: *ref. cat* Reference category

The results of the first analysis presented in Table 2 were confined to patients residing in NYS and the results of the second analysis also displayed in Table 2 were limited to just residents of NYC. The tables present the unstandardized *b* coefficients, the exponentiated *b* coefficients (the rate ratios) the significance levels of the coefficients, and the 95% CIs of the rate ratios.

Inspection of the data for NYS reveals that the year cubed term was statistically significant, denoting the presence of a cubic fit concerning the time variable. This result indicates that, after holding constant the demographic variables in the model, the likelihood of being injured changed direction twice with the passage of time.

Consistent with the findings from earlier research, there is a noticeable gender gap in the likelihood of sustaining a scooter injury. Males are one and a half times as likely to visit an ED as a result of a scooter injury than females.

As expected, age is a major determinant of the risk of injury. Compared to patients 45 years of age and older (the reference category), individuals in the under 5 years of age category are about 10 times more likely to incur a scooter injury. This ratio becomes even more pronounced among the age group 5 to 9 (44.2:1) and the age group 10 to 14 (28.2:1). The data further show that non-Hispanic Whites and non-Hispanic Blacks have a greater probability of being injured than Hispanics (the reference category). Non-Hispanic Asians, on the other hand, have a significantly lower probability of being injured than Hispanics.

The results for NYC adhere to the same general pattern as found for NYS. Again, the year cubed term is statistically significant. The results for NYC also closely correspond to the results for NYS with regards to the effects of gender and age. Again, males and individuals in the age groups 5 to 9 and 10 to 14, were far more likely to sustain an injury than their counterparts. On the other hand, the odds of being injured by non-Hispanic Whites and non-Hispanic Blacks were not significantly different than the odds for Hispanics, as was found in the data for NYS.

### Local analysis: New York City

Table 3 displays the relationship between key sociodemographic variables and the rate of injuries from nonmotorized scooters at the neighborhood level in NYC. Neighborhood is defined by the 42 United Health Fund districts in the City. The data show that the injury rate is positively associated with the percent of the population which is either non-Hispanic White or the percent which is non-Hispanic Asian. Oppositely, the percent of the population which is non-Hispanic Black or the percent which is Hispanic are negatively correlated with the injury rate.

**Table 3:** Correlations Between Demographic Characteristics and Nonmotorized Scooter Injury Rate in New York City United Health Fund Districts (*N* = 42)

Demographic Characteristic	Correlation	
	Coefficient	*p* Value
Percent non-Hispanic White^b^	.45	.002
Percent non-Hispanic Black^b^	-.48	.001
Percent non-Hispanic Asian^b^	.44	.003
Percent Hispanic^b^	-.35	.023
Median family income^bc^	.59	.000
Per capita income^bc^	.61	.000
Percent of population 25 years or age or older who have a B.A. degree or more^b^	.61	.000
Percent of population under 18 below the poverty rate^b^	-.40	.009
Percent of population with no health insurance^b^	-.21	.171
Percent of insured population with public health insurance^b^	-.54	.000
Number of major skate parks^b^	-.14	.368

^b^Analysis is confined to those under the age of 18

^c^Calculated by computing the median value of this variable for all zipcodes within each UHF district

On the series of variables measuring economic status, a consistent finding emerges: the injury rate tends to *go up* with increases in the income level or educational attainment of the neighborhood’s inhabitants. Median family income, per capita income, and the percent of the population over 25 with a B.A. degree or more are all positively related to the injury rate. Additionally, the percent of the population under 18 below the poverty rate, the percent of the population without health insurance, and the percent with health insurance which is public are all negatively associated with the injury rate. The relationship between the number of skate parks and the injury rate was negligible (*r* = -0.14).

### Spatial distribution of scooter injuries in New York City’s neighborhoods

**Fig. 2:**
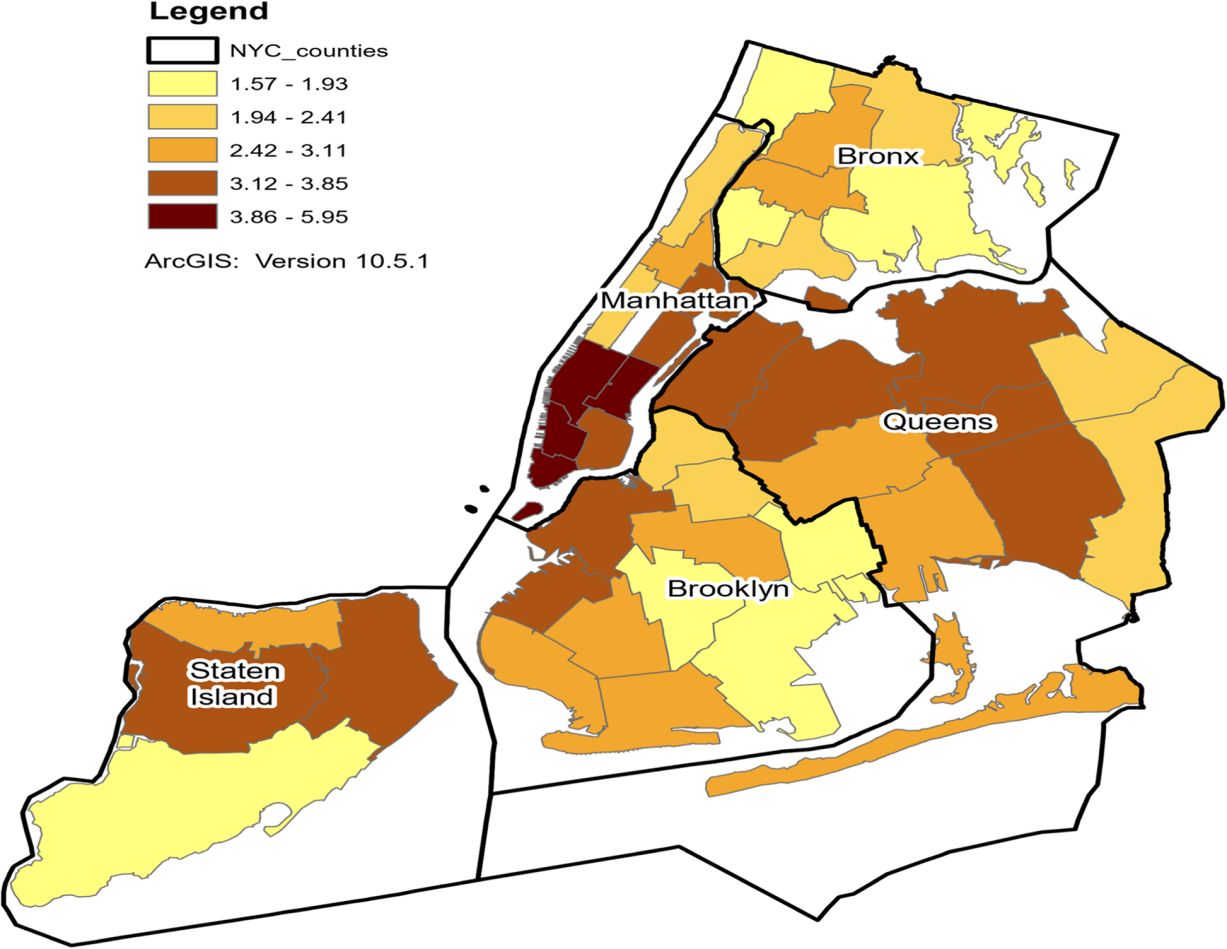
Map of Injury Rates by UHF Districts in New York City

Figure 2 presents a choropleth map of the injury rates by UHF districts in NYC. The rates were calculated by first averaging the number of scooter injuries sustained by patients under the age of 18 in 2018, 2019, and 2020 in each UHF district. This step was undertaken to obtain a more stable measure of injuries than would have been obtained by relying on the number of injuries for a single year. Next these averages were divided by the number of inhabitants under the age of 18 in each UHF district and then multiplying this ratio by 10,000.

The map shows that the injury rates were not uniformly distributed across the UHF districts. In particular, certain contiguous neighborhoods in the southern tip of Manhattan had noticeably higher rates than other UHF districts. These neighborhoods included the following: Chelsea-Clinton, Gramercy Park-Murray Hill, Greenwich Village-Soho, and Lower Manhattan. Importantly, these same neighborhoods have also been identified in other research as having relatively high rates of pedestrians injured in collisions with cyclists [9]. A Global Moran’s I yielded a Index value of 0.45 (*p* < 0.001) indicating a pattern of spatial clustering.

To investigate further the reasons why the rate of scooter injuries was markedly higher in certain neighborhoods in southern Manhattan, an additional analysis was conducted examining the frequency distribution of places of injury (e.g., home, place of recreation and sport, street/highway, etc.) within each of the UHF districts. Since the “place of injury” variable was not available for the SPARCS data starting in 2016, the analysis rested on the “place of injury” variable for the SPARCS data years spanning 2011 to 2015. The analysis revealed that a higher proportion of scooter injuries occurred in the streets or highways in the neighborhoods in southern Manhattan than the average proportion of injuries occurring in the streets and highways for all neighborhoods. However, the overall correlation between the injury rate and the proportion of injuries taking place in the streets or highways at the neighborhood level was not significant.

## Discussion

This study has tracked the rate of injuries resulting from the use of nonmotorized scooters over the time period from 2005 to 2020 in NYS and NYC. The study has produced evidence that the injury rate in both geographic areas has declined substantially in recent years.

One factor which clearly contributed to the observed decline in scooter injuries in NYS and NYC was the change in the coding system from ICD-9-CM to ICD-10-CM. In both NYS and NYC this study noted a precipitous decline in the rates of scooter injuries immediately after 2015. This same time period coincided with the transition from ICD-9-CM to ICD-10-CM. Other research has also documented the immediate impact of transitioning from ICD-9-CM to ICD-10-CM on injury trends but the impact was more fleeting [10].

After 2016 there was no noticeable increase in the injury rate in NYS but a marked increase in the rate in NYC. One possible explanation for these disparate trends was that the popularity of nonmotorized scooters in NYC as a recreational vehicle – particularly in the pandemic year of 2020 when school children were isolated at home – was greater in the NYC than elsewhere in the State.

In addition to the change in the coding scheme, other explanations could be posited to account for the decrease in the injury rate. These possible explanations include the following: (1) the greater use of protective gear such as helmets and knee and elbow pads, (2) the more sedentary lifestyle of younger children, and (3) a shift from using nonmotorized scooters to motorized scooters among older children and adults.

Though this study has documented a decrease in scooter injuries prior to 2016, it should be kept in mind that the number of annual scooter injuries is still sizable and appears to be growing in the most recent time interval. According to estimates based on data furnished by the United States Consumer Product Safety Commission, the number of nationwide injuries totaled 45,376 in 2019 [1]. Moreover, the data for both NYS and NYC showed there has been a growth in the rate of injuries since 2018. Particularly, in NYC in 2020 the rate of injuries soared. It is likely that the advent of the coronavirus pandemic in 2020, during which many children were sequestered at home, spurred a greater interest in nonmotorized scooters. This, in turn, may account for the higher injury rate in 2020.

Along with analyzing trends and demographics at the NYS and NYC levels, this study examined the socio-economic characteristics associated with scooter injury rates at the neighborhood level in NYC. The data showed that injury rates were positively correlated with a number of socio-economic variables at the neighborhood level. One possible reason for this finding is that riding a push scooter might be a more popular recreational activity in more affluent neighborhoods. Another possible reason may be based on the price of purchasing a push scooter.

This study also produced a thematically-shaded map of the injury rates at the neighborhood level in NYC. The map revealed a clustering of neighborhoods with higher than average injury rates. One geographic area, in particular, which comprised contiguous neighborhoods with higher than average injury rates was the southern tip of Manhattan. Notably, previous research has found that these same neighborhoods were characterized by a disproportionately large number of pedestrians who were injured in collisions with cyclists [9]. Moreover these same neighborhoods were observed to have a comparatively greater incidence of scooter injuries occurring in the streets as opposed to other places. These disparate findings suggest that the street environment in these neighborhoods poses certain hazards for scooter riders or pedestrians. Hazardous conditions might include uneven street pavement, sidewalk cracks, or inadequate infrastructure for all types of street users. Further research needs to be conducted to identify the specific factors in this environment responsible for the elevated injury rates of scooter riders and other street users.

### Limitations

Two limitations of this study pertain to the database upon which this study rests – patient-level records from ED visits in NYS and NYC. First, the database excludes individuals who may have pursued treatment in alternative venues such as a private physician’s office or an urgent care center. Graphs depicting the annual rates of patients who were hospitalized as a result of their injuries in both NYS and NYC adhere to the same general patterns found for annual rates for outpatients in these two areas. This finding tends to bolster the representativeness of the patients included in this study. Second, patients who sustained injuries riding a motorcycle (which requires a license) may have reported their injuries as owing to riding a nonmotorized scooter. The bias resulting from patients’ misrepresenting the cause of their injuries is difficult to measure. However, the age distribution of the injured individuals reported in this study which skews heavily towards patients 14 years of age and younger suggests that this bias would not be a serious one. Also it is reasonable to assume that this bias would not change greatly over time and therefore would not account for variation in temporal patterns.

## Conclusion

This study has found that injuries from nonmotorized scooters have spiraled downwards in NYS and NYC in the past decade. Recently, though, there has been an uptick in the number of scooter-related injuries in NYC. Young children, especially those in the 5 to 9 and 10 to 14 year old age groups, are particularly vulnerable to being injured.

This study has also mapped the incidence of injuries within different neighborhoods in New York City. The map revealed a concentration of injuries in certain neighborhoods. These same neighborhoods also have been characterized as being hazardous to other street users such as pedestrians. Identifying the specific factors operating in these neighborhoods which contributed to the elevated number of injuries by scooters can increase our understanding of the causes of these injuries and hopefully lead to a reduction in their number.

## Data Availability

The datasets for this study are derived from three main sources: 1) Statewide Planning and Research Cooperative System (SPARCS) data available at https://www.health.ny.gov/statistics/sparcs, 2) CDC Wonder Bridged-Race Population Estimates available at https://wonder.cdc.gov/bridged-race-pulation.html, and 3) American Community Survey (ACS) 2015–2019 (5-Year Estimates) available at https://www.socialexplorer.com/explore-tables. To access and use the SPARCS data, approval must be secured from this agency. The other two sources are open accessed.

## Abbreviations

CDC: Centers for Disease Control and Prevention
ED: Emergency Department
ICD: International Classification of Diseases
NYC: New York city
NYS: New York state
SPARCS: Statewide Planning and Research Cooperative System
UHF: United Health Fund
